# Diffuse Idiopathic Skeletal Hyperostosis (DISH): Potential Links Between Gut Microbiota and Spinal Degenerative Diseases

**DOI:** 10.3390/nu18193168

**Published:** 2026-09-26

**Authors:** Iddrisu Ibrahim, Kelci Lawrence, Othreniel Angel Forte, Latrell Huitt, Navitri Chandra Naidu, Nathaniel Ajibola, Junhuan Xu, Robertson K. Boakai, Fortune Akabanda, James Owusu-Kwarteng, Olufemi S. Ajayi, Joseph Atia Ayariga

**Affiliations:** 1 The Microbiology Program, College of Science, Technology, Engineering, and Mathematics (C-STEM), Alabama State University, Montgomery, AL 36104, USA; iiddrisu3715@alasu.edu (I.I.); klawrence0670@alasu.edu (K.L.); oforte3773@alasu.edu (O.A.F.); lhuitt@alasu.edu (L.H.); nnaidu@alasu.edu (N.C.N.); najibola7327@alasu.edu (N.A.); jxu@alasu.edu (J.X.); brobertson@alasu.edu (R.K.B.); 2 The Industrial Hemp Program, College of Science, Technology, Engineering, and Mathematics (C-STEM), Alabama State University, Montgomery, AL 36104, USA; 3 African Drug Discovery and Innovation Network, Sunyani P.O. Box AB45, Ghana; james.owusu-kwarteng@uenr.edu.gh; 4 Department of Food Science and Technology, University for Development Studies, Tamale P.O. Box TL1350, Ghana; 5 Department of Food Science and Technology, School of Agriculture and Technology, University of Energy and Natural Resources, Sunyani P.O. Box 214, Ghana

**Keywords:** DISH, gut microbiota, inflammatory bowel disease, intervertebral disc degeneration, irritable bowel syndrome, skeletal hyperostosis, spondylitis

## Abstract

Diffuse Idiopathic Skeletal Hyperostosis (DISH) is a systemic, non-inflammatory skeletal disorder characterized by progressive calcification and ossification of spinal ligaments and entheses. Its strong associations with aging, obesity, type 2 diabetes, and metabolic syndrome raise the possibility that metabolic and inflammatory pathways may contribute to disease development. The gut microbiota participates in nutrient metabolism, short-chain fatty acid production, bile-acid transformation, intestinal-barrier maintenance, and immune regulation; these functions provide a plausible framework for examining whether intestinal dysbiosis may influence spinal and musculoskeletal tissues. This narrative review therefore evaluates the potential relationship between gut microbiota, DISH, and related spinal degenerative diseases. It considers the nutritional, metabolic, immunological, and host-interaction functions of the gut microbiota before examining proposed gut–spine, gut–bone, gut–joint, gut–disc, gut–ligament, and gut–muscle pathways. Particular attention is given to the apparent paradox that most microbiome–bone evidence concerns osteoporosis and bone loss, whereas DISH is characterized by excessive ectopic bone formation. Differences in short-chain fatty acid concentration, receptor expression, local versus systemic signaling, and osteoblast–osteoclast microenvironments may produce divergent skeletal effects, but these mechanisms have not been tested directly in DISH. Recent studies support associations between selected microbial taxa and intervertebral disc degeneration or spinal stenosis; however, direct clinical evidence linking gut microbiota to DISH remains limited. Consequently, dietary modification, probiotics, prebiotics, and fecal microbiota transplantation should be regarded as research considerations rather than established treatments for DISH. Well-characterized case–control and longitudinal studies integrating microbiome profiling, metabolomics, inflammatory markers, and serial imaging are required to determine whether reproducible microbial or metabolite signatures precede DISH onset or progression. The pathways discussed in this review are therefore presented as biologically plausible, testable hypotheses and not as established causal relationships.

## 1. Introduction

Hyperostosis denotes excessive formation or thickening of bone. Its distribution and clinical consequences vary according to the affected site and the underlying disorder. Diffuse idiopathic skeletal hyperostosis (DISH), also known as Forestier disease, is a systemic enthesopathic disorder characterized by calcification and ossification of ligaments and entheses, most conspicuously along the anterolateral spine [1,2,3,4]. Under the classic Resnick and Niwayama criteria, radiographic diagnosis requires flowing ossification across at least four contiguous vertebral bodies, relative preservation of intervertebral-disc height, and absence of extensive apophyseal-joint ankylosis or sacroiliac erosion typical of ankylosing spondylitis [5,6]. Newer imaging studies also describe early-phase disease, but these observations have not removed the need to distinguish DISH from degenerative spondylosis, axial spondyloarthritis, and ossification of the posterior longitudinal ligament. DISH is often asymptomatic; when clinically apparent, it may cause spinal stiffness, reduced range of motion, back or peripheral entheseal pain, dysphagia from cervical osteophytes, difficult airway management, or neurological compression. The ankylosed spine is also susceptible to unstable fractures after low-energy trauma, and delayed recognition can result in neurological deterioration [3,4,5]. Management is therefore directed to the individual clinical problem and may include analgesia, physical activity and mobility programs, metabolic-risk management, fracture precautions, and surgery for selected fractures or severe compressive complications [5,6,7]. The cause of DISH remains uncertain. Age, male sex, obesity, type 2 diabetes, hyperinsulinemia, dyslipidemia, and other metabolic features are repeatedly associated with the disorder, while genetic and local mechanical factors may modify susceptibility [3,4,8]. These associations support investigation of pathways that favor entheseal osteogenesis and ligament ossification, but they do not establish that any single metabolic exposure causes DISH. The present review examines whether gut-microbiota-related changes in microbial metabolites, intestinal-barrier function, immune signaling, and host metabolism could contribute to this pro-osteogenic environment. Because direct microbiome studies in DISH remain scarce, evidence from metabolic disease, intervertebral-disc degeneration, osteoporosis, and related musculoskeletal conditions is treated as indirect and hypothesis-generating rather than as proof of a gut-microbiota–DISH axis.

## 2. Clinical Definition, Differential Diagnosis, and Complications

The Resnick and Niwayama criteria remain the most widely used radiographic framework for diagnosing DISH. They require flowing calcification and ossification along the anterolateral aspect of at least four contiguous vertebral bodies, relative preservation of intervertebral disc height, and absence of extensive apophyseal-joint ankylosis or sacroiliac-joint erosion, sclerosis, or fusion [9]. These criteria primarily identify established disease; more recent longitudinal imaging studies indicate that DISH develops along a continuum from isolated or incomplete bridging to mature flowing ossification [10]. This distinction is important when early-stage disease is considered in epidemiological or mechanistic studies.

DISH must also be distinguished from ossification of the posterior longitudinal ligament (OPLL). DISH predominantly involves the anterior longitudinal ligament and entheses, producing anterolateral bridging, whereas OPLL affects the posterior longitudinal ligament and may directly narrow the spinal canal, thereby increasing the risk of myelopathy. The two disorders may coexist, particularly in the cervical spine, but they are not interchangeable and may have different neurological and surgical implications [11].

The clinical importance of DISH extends beyond pain and stiffness. The ankylosed spine behaves as a long lever arm and may sustain unstable fractures after low-energy trauma, sometimes with delayed displacement or neurological deterioration [12,13]. Cervical anterior osteophytes may also compress or displace the pharynx and esophagus, causing dysphagia and, less commonly, airway symptoms [14,15]. These complications make accurate recognition of DISH clinically important even when the underlying disease is initially asymptomatic.

## 3. Metabolic Context and Rationale for Microbiome Investigation

Although the cause of DISH remains uncertain, obesity, type 2 diabetes, insulin resistance, dyslipidemia, and metabolic syndrome are repeatedly associated with the disorder [4,14,16,17]. These associations are relevant to microbiome research because the same metabolic conditions are accompanied by changes in diet, microbial composition, intestinal permeability, bile-acid metabolism, and systemic inflammatory tone. This overlap provides a rational basis for investigating a gut–metabolic–enthesis pathway, but it does not establish that dysbiosis initiates or drives ectopic ossification in DISH.

Accordingly, this review preserves the broader nutritional, metabolic, and immunological background needed to understand the proposed mechanisms, while consistently separating direct DISH evidence from evidence extrapolated from osteoporosis, osteoarthritis, intervertebral disc degeneration, metabolic disease, or experimental models. The principal question is not whether the gut microbiota affects human physiology in general, but whether any reproducible microbial or metabolite pattern is specifically associated with DISH onset, progression, pain, or complications.

## 4. Literature Search Strategy

This narrative review was developed through focused searches of PubMed and Google Scholar from database inception to September 2023 and was updated during revision through September 2026. Search terms included combinations of “diffuse idiopathic skeletal hyperostosis”, “DISH”, “gut microbiota”, “microbiome”, “spinal degeneration”, “intervertebral disc”, “bone remodeling”, “short-chain fatty acids”, “inflammation”, “obesity”, “type 2 diabetes”, and “metabolic syndrome”. English-language clinical, epidemiological, animal, and mechanistic studies relevant to the proposed gut–musculoskeletal pathways were considered. Reference lists of relevant articles were also screened. Because the available studies were heterogeneous and direct DISH-specific microbiome evidence was limited, the findings were synthesized narratively rather than quantitatively.

## 5. Gut Microbiota (GM)

The gut microbiota is the community of bacteria, archaea, fungi, viruses, and other microorganisms inhabiting the gastrointestinal tract. Bacteria usually dominate sequence-based surveys, but non-bacterial members also contribute to community structure and function. Taxonomic composition varies along the gastrointestinal tract and among individuals, while microbial genes collectively expand the range of dietary and endogenous compounds that can be metabolized [18,19].

Functions attributed to the gut microbiota include fermentation of otherwise poorly digestible carbohydrates, production of short-chain fatty acids and selected vitamins, transformation of bile acids and xenobiotics, colonization resistance, and interaction with epithelial and immune cells (Figure 1) [20,21]. These functions are distributed across microbial communities rather than confined to a universally beneficial set of species. Their effects depend on microbial strain, substrate availability, intestinal location, host physiology, and disease context. The microbiota also interacts with mucus and tight-junction regulation, but intestinal-barrier integrity is jointly determined by epithelial, immune, dietary, and microbial factors.

Altered gut-microbial composition or function has been reported in gastrointestinal, metabolic, immune-mediated, and neuropsychiatric disorders [22]. The term dysbiosis is commonly used for these changes, but it does not denote one reproducible community pattern across diseases. Cross-sectional associations cannot determine whether a microbial difference precedes disease, results from disease, or reflects diet, medication, geography, age, or other confounders.

Diet is a major modifiable determinant of gut-microbial structure and metabolism. Fiber-rich dietary patterns provide fermentable substrates, whereas low-fiber and highly processed dietary patterns alter substrate availability and may favor different metabolic functions. Antibiotics can markedly disturb susceptible taxa, and non-antibiotic medicines can also alter microbial composition or activity. Bidirectional signaling through neural, endocrine, immune, and metabolic routes is often described as the gut–brain axis [23,24]. Associations between microbial features and mental-health phenotypes are being investigated, but they do not establish a simple microbial cause of depression, anxiety, or behavior.

Interventions that modify the gut microbiota include dietary change, prebiotics, probiotics, and fecal microbiota transplantation (FMT) [25]. FMT transfers screened donor stool or a microbiota-based preparation to the gastrointestinal tract of a recipient. Its best-established clinical role is the prevention of recurrent Clostridioides difficile infection; applications in metabolic, immune, neurological, or musculoskeletal disorders remain investigational. Microbiome sequencing can describe community composition and functional potential, but interpretation is affected by diet, medication exposure, geography, age, sampling, and analytical methods. Consequently, a microbiota profile cannot yet be used alone to diagnose DISH or select a DISH-specific treatment [26].

Microbial metabolism has been associated with obesity, type 2 diabetes, metabolic syndrome, irritable bowel syndrome, and inflammatory bowel disease [18]. These conditions differ in pathophysiology, and no single “unhealthy microbiota” explains them. In metabolic disease, relevant mechanisms may include altered SCFA and bile-acid profiles, barrier dysfunction, endotoxin exposure, and interactions with insulin and inflammatory signaling. In gastrointestinal disease, local inflammation, motility, medication, and diet can themselves reshape the community. The same caution applies to gut–brain findings: microbial differences may be causal, consequential, or confounded.

Relevance to DISH. The metabolic and immune functions described above are relevant because DISH frequently coexists with obesity, type 2 diabetes, and metabolic syndrome. Nevertheless, these shared associations remain indirect. A microbiome feature associated with obesity or intestinal inflammation cannot be assumed to be specific to DISH, and future studies must distinguish disease-specific signals from the effects of age, diet, medication, and metabolic comorbidity [17].

## 6. Gut Microbiota and Nutrition

Bresser et al. [27] reviewed the relationship between gut microbiota and nutrition. Microbial enzymes extend the host’s capacity to metabolize dietary fibers, resistant starches, polyphenols, and other compounds that reach the colon. In turn, dietary composition determines which substrates are available for microbial growth and metabolite production.

Fiber-rich foods, including fruits, vegetables, legumes, and whole grains, provide substrates that many gut microorganisms ferment to short-chain fatty acids (SCFAs), including acetate, propionate, and butyrate [28]. Prebiotics are substrates selectively used by host microorganisms and may support a health-associated microbial community; examples include inulin and fructo-oligosaccharides. A 2022 review described the mechanisms through which prebiotics are metabolized and their potential effects on metabolic health [29]. Probiotics are live microorganisms that confer a health benefit when administered in adequate amounts, but their effects are strain- and indication-specific. Fermented foods such as yogurt, kefir, sauerkraut, and kimchi may contain live microorganisms and fermentation products, although not every fermented food meets the definition of a probiotic [30].

Dietary diversity can increase the range of substrates available to the gut microbiota, whereas highly processed, low-fiber dietary patterns may reduce fermentation of complex plant carbohydrates. Microbiota composition has been associated with obesity and metabolic disorders, but sequencing alone does not yet provide validated, individualized dietary prescriptions [31]. Nutritional interventions may be clinically useful in disorders such as irritable bowel syndrome or inflammatory bowel disease, although the choice of diet depends on the diagnosis, nutritional adequacy, tolerability, and supporting evidence rather than on a generic goal of “balancing” the microbiota [21].

In the context of DISH, nutrition may therefore act through several overlapping routes: body weight and insulin sensitivity, the availability of substrates for microbial fermentation, vitamin and mineral absorption, and systemic inflammatory signaling. These routes justify investigation, but current evidence does not show that a particular diet prevents or reverses the characteristic ligamentous ossification of DISH.

## 7. Gut Microbiota and Metabolism

### 7.1. Microbial Fermentation and Energy Metabolism

Microbial fermentation extends the host’s capacity to use dietary carbohydrates that escape digestion in the small intestine. In the colon, microbial communities metabolize these substrates and generate SCFAs, principally acetate, propionate, and butyrate [32]. The host absorbs most SCFAs: colonocytes preferentially oxidize butyrate, whereas acetate and propionate enter portal and peripheral metabolism. SCFAs also act as signaling molecules that can influence epithelial-barrier integrity, immune-cell activity, glucose and lipid metabolism, and appetite regulation. Their concentrations and effects depend on dietary substrate availability, community composition, intestinal transit, absorption, and host metabolism; they should therefore not be interpreted simply as an additional energy store or as uniformly beneficial metabolites [33].

### 7.2. Metabolic Efficiency

The gut microbiota contributes to metabolic efficiency by fermenting complex carbohydrates and dietary fibers that are not fully digested by human enzymes [34]. The resulting SCFAs can be absorbed and used in host metabolism, while also participating in receptor-mediated and epigenetic signaling. Diet-associated changes in microbial community structure may alter the amount and relative proportions of SCFAs and other metabolites. Observational and experimental studies have linked microbial dysbiosis with obesity and metabolic dysfunction, but the idea that particular bacteria cause weight gain merely by extracting more calories from the same food is an incomplete explanation. Energy intake, diet quality, host genetics, medication use, physical activity, and adipose-tissue and endocrine responses remain important determinants [35].

### 7.3. Appetite Modulation

Appetite regulation integrates hypothalamic, gastrointestinal, adipose-tissue, sensory, behavioral, and environmental signals. Microbial metabolites may modify enteroendocrine signaling and the release or action of hormones involved in hunger and satiety, but the magnitude and clinical relevance of these effects vary across experimental systems [36].

Leptin is produced mainly by adipose tissue and signals information about energy stores to the brain. Obesity is often accompanied by high circulating leptin and reduced leptin responsiveness rather than a simple deficiency of the hormone [37]. Ghrelin, produced largely in the stomach, participates in meal initiation and energy balance. Diet and microbial metabolites may influence these hormonal pathways, but evidence does not support assigning food cravings or overeating to the preferences of particular gut bacteria. Appetite and body weight remain jointly determined by diet, energy expenditure, host genetics, medication, sleep, psychological factors, and the wider food environment [36].

### 7.4. Glucose and Lipid Metabolism

Gut microorganisms can modify dietary lipids, bile acids, and host lipid signaling. Dysbiosis has been associated with altered triglyceride and cholesterol profiles, but these relationships are influenced by diet, adiposity, liver metabolism, genetics, and medication use [38]. Microbial composition should therefore be considered one component of lipid metabolism rather than a direct measure of cardiovascular risk.

Gut-microbial features and metabolites have also been associated with glucose regulation and insulin sensitivity [39]. Proposed mechanisms include barrier dysfunction, endotoxin exposure, SCFA and bile-acid signaling, and interactions with adipose tissue and hepatic metabolism. These pathways may contribute to insulin resistance in susceptible hosts, but microbiome associations do not replace established determinants such as adiposity, diet, physical activity, genetics, and medication exposure [21].

### 7.5. Metabolism and Inflammation

Metabolic dysfunction and chronic low-grade inflammation frequently coexist. In obesity and metabolic syndrome, adipose-tissue inflammation, altered intestinal permeability, circulating microbial products, and host immune responses may interact [40]. A changed microbiota can contribute metabolites or molecular patterns that modify inflammatory signaling, while inflammation, diet, medication, and altered transit can reshape the microbiota. Insulin resistance is one clinically important outcome of this network, but it is not attributable to a single microbial pattern [41].

These metabolic pathways are especially pertinent to DISH because insulin resistance, central adiposity, dyslipidemia, and related metabolic disturbances recur in clinical cohorts. The most defensible interpretation is that the gut microbiota may participate in a wider metabolic environment that could influence skeletal remodeling. Whether it contributes independently after adjustment for obesity, diabetes, medication use, and physical activity remains unknown [17].

### 7.6. Short-Chain Fatty Acids

Short-chain fatty acids are products of microbial fermentation with metabolic and signaling functions. Butyrate is particularly relevant to the colonic epithelium because it is a preferred energy substrate for colonocytes and can support epithelial-barrier integrity. It may influence tight-junction proteins, mucus production, immune-cell differentiation, and inflammatory signaling, although the direction and magnitude of these effects depend on concentration, tissue context, and disease state [42]. Common SCFAs and the in silico biotransformations evaluated in this review are summarized in Table 1. These predictions describe possible enzymatic products and do not demonstrate that the reactions occur in the human gut or that the products have clinical effects. Reduced butyrate availability has been associated with impaired epithelial function in several experimental settings, but disease-specific interpretation requires direct measurement of microbial function and host exposure [43].

## 8. Biotransformation of Common Short-Chain Fatty Acids

Dietary fibers and other fermentable substrates provide major carbon sources for saccharolytic members of the gut microbiota. Their fermentation generates SCFAs, of which acetate, propionate, and butyrate commonly account for approximately 90–95% of the SCFAs detected in the colon. The amount and relative proportions produced vary with substrate composition, microbial community structure, intestinal transit, absorption, and cross-feeding among microorganisms. Gut and host enzymes may further transform SCFAs, but the occurrence, abundance, and physiological significance of the predicted products require experimental confirmation. Table 1 summarizes the predominant SCFAs considered here and their predicted transformations by gut microbial enzymes.

Butyrate can inhibit histone deacetylases and signal through G-protein-coupled receptors, thereby influencing epithelial-cell proliferation, differentiation, apoptosis, and immune responses. In cell and animal models, these mechanisms have been studied in relation to colorectal carcinogenesis and neuroinflammation [44]. Their effects are concentration- and context-dependent; results from experimental exposure cannot be translated directly into cancer prevention, cognitive benefit, or treatment of depression and anxiety. Dietary fiber can increase colonic fermentation, but individual responses depend on microbial capacity, transit, disease state, and total dietary pattern. Claims that butyrate or a “balanced microbiota” broadly improves health therefore require outcome-specific clinical evidence [42,45,46].

Interpretive limitation. The reactions listed in Table 1 are computational predictions and were not validated experimentally. They should not be treated as evidence that these products are formed in the human gut, reach the circulation, or influence DISH. Establishing biological relevance would require targeted metabolomic detection, enzyme-level confirmation, concentration measurements, and functional testing in appropriate cell, animal, or clinical models.

### 8.1. Bile-Acid Metabolism

The gut microbiota modifies bile-acid metabolism. Primary bile acids synthesized in the liver are conjugated and secreted into the small intestine, where they facilitate digestion and absorption of dietary lipids [47]. Microbial bile-salt hydrolases and other enzymes deconjugate and transform bile acids, generating secondary bile acids with different physicochemical and signaling properties. In turn, bile acids shape microbial growth through detergent and antimicrobial effects [48].

Bile acids emulsify dietary fats and support absorption of lipids and fat-soluble vitamins. Beyond digestion, they activate receptors including farnesoid X receptor and Takeda G-protein-coupled receptor 5, which participate in glucose, lipid, and energy metabolism. Changes in the bile-acid pool may therefore influence metabolic phenotypes, but their effects depend on hepatic synthesis, intestinal transit, microbial transformation, and enterohepatic circulation [49,50].

### 8.2. Body Weight and Composition

Gut-microbial composition differs on average between some lean and obese study populations, but the findings are heterogeneous and strongly influenced by diet, geography, medication, and study methods [51]. Early models emphasized increased microbial energy harvest; subsequent work indicates that obesity-associated microbiome effects are more complex and may involve substrate use, bile acids, SCFAs, barrier integrity, and inflammatory signaling [52].

Microbial fermentation converts otherwise poorly digestible substrates into SCFAs that contribute a modest amount of host energy and participate in metabolic signaling [53]. Dysbiosis may also accompany barrier dysfunction and low-grade inflammation. These pathways can interact with appetite and energy expenditure, but they do not imply that a specific microbial community inevitably causes weight gain. Interpretation must account for total energy intake, diet quality, adiposity, physical activity, host genetics, and medication exposure [37].

### 8.3. Drug Metabolism

Gut microorganisms can activate, inactivate, or alter the toxicity of some drugs through reduction, hydrolysis, deconjugation, and other enzymatic reactions [54]. Microbial metabolism can also affect enterohepatic recycling or interact with host drug-metabolizing enzymes and transporters. The direction and clinical magnitude are drug-specific; competition for nutrients or ecological space is not a general explanation of drug bioavailability. Medication use is therefore both a potential microbiome modifier and an important confounder in observational microbiome studies [55].

### 8.4. Metabolic-Health Interventions

Probiotics, prebiotics, dietary interventions, and FMT are being studied as methods of modifying the gut microbiota in metabolic disease. Their clinical evidence and regulatory status differ substantially. FMT is a specialized procedure in which screened donor material or a microbiota-based preparation is delivered to a recipient [56]. Its established therapeutic use is concentrated in recurrent Clostridioides difficile infection. Trials in obesity, insulin resistance, and other metabolic disorders have produced variable findings and have not established FMT as routine metabolic therapy. Donor selection, pathogen screening, route of administration, durability of engraftment, and adverse-event surveillance are essential considerations. No microbiota-directed intervention has been shown to prevent or treat DISH; any such application would require disease-specific preclinical evidence followed by controlled clinical trials [57,58].

Taken together, microbial effects on bile-acid metabolism, body composition, drug transformation, and metabolic interventions provide a mechanistic bridge to the metabolic phenotype of DISH. However, probiotics, prebiotics, or FMT have not been shown to modify DISH progression, and these approaches should not presently be described as established preventive or therapeutic strategies.

## 9. Gut Microbiota and Immunomodulation

### 9.1. Immune System Development

The gut microbiota contributes to immune-system development, particularly during early life. Microbial colonization supplies antigenic and metabolic signals that support maturation of mucosal lymphoid tissues, regulatory T-cell responses, immunoglobulin production, and discrimination between harmful organisms and tolerated commensals [59]. This process is shaped by delivery mode, feeding, antibiotic exposure, infection, diet, and the surrounding environment. Microbial exposure does not simply “strengthen” immunity; it helps calibrate inflammatory and tolerogenic responses. Experimental and clinical observations associate disrupted early microbial development with altered immune phenotypes and some allergic or inflammatory disorders, but the effects are context-dependent and do not imply that a single microbial pattern determines later disease [60].

### 9.2. Immune Tolerance

The concept of immune tolerance is fundamental to the proper functioning of the immune system. Immune tolerance refers to the ability of the immune system to recognize and tolerate harmless substances, such as allergens or the body’s own tissues, without mounting an unnecessary or harmful immune response. This state of balance is crucial for preventing immune overreactions, allergies, and autoimmune conditions, where the immune system mistakenly attacks the body’s own cells and tissues [61,62]. The presence of diverse gut microbes helps educate immune cells, teaching them to differentiate between beneficial microorganisms and potentially harmful invaders. This education enables immune cells to avoid attacking beneficial microbes and remain tolerant to them. A well-balanced gut microbiota helps regulate inflammation within the gut and the body as a whole. Inflammation is a fundamental immune response, but when it is excessive or chronic, it can lead to tissue damage and autoimmune conditions. The gut microbiota helps maintain the appropriate balance of inflammation, preventing unnecessary immune responses against harmless substances. Healthy and diverse gut microbiota act as a protective barrier against harmful pathogens, preventing them from establishing themselves in the gut. By inhibiting the growth of pathogens, the gut microbiota reduces the need for a heightened immune response [63,64].

### 9.3. Inflammatory Regulation

Gut microorganisms can influence inflammatory regulation through SCFAs, bile-acid derivatives, microbial molecular patterns, and effects on the epithelial barrier [65]. Butyrate and related metabolites may alter regulatory T-cell differentiation and cytokine production in experimental systems. Conversely, barrier disruption can increase host exposure to microbial products and amplify immune activation. These mechanisms are relevant to inflammatory bowel disease and metabolic inflammation, but “anti-inflammatory bacteria” is an oversimplification: effects depend on strain, metabolite concentration, host context, and tissue compartment [66,67].

### 9.4. Protection Against Pathogens

Resident microorganisms contribute to colonization resistance by occupying ecological niches, competing for nutrients and attachment sites, modifying local pH and metabolites, and producing bacteriocins or other inhibitory compounds [68,69]. These effects are community- and pathogen-specific. Protection can be weakened by antibiotics, infection, dietary change, or barrier injury, but the presence of a taxon conventionally described as beneficial does not by itself establish resistance to a pathogen.

### 9.5. Relevance to DISH

For DISH, the potential relevance of these immune pathways lies in their interaction with metabolic inflammation and tissue remodeling rather than in evidence of a classic autoimmune disease. None of the cytokine patterns in Table 2 has been validated as a DISH-specific biomarker, and the cytokine associations observed in intestinal or autoimmune disorders should not be transferred directly to ligament or enthesis ossification.

## 10. Gut Microbiota–Host Interactions

Gut microbiota–host interactions involve nutrient metabolism, epithelial-barrier maintenance, colonization resistance, immune regulation, and microbial signaling. Fermentation of otherwise poorly digestible carbohydrates generates SCFAs and other metabolites that can be absorbed or sensed by host cells [66]. Butyrate supports colonocyte metabolism, while acetate and propionate participate in hepatic and peripheral pathways. Microorganisms also transform bile acids, amino acids, xenobiotics, and dietary compounds, thereby altering the molecules presented to intestinal and systemic tissues [22]. The intestinal barrier limits translocation of microbes and microbial products, and the resident community competes with pathogens for nutrients and ecological niches. In parallel, microbial antigens and metabolites help calibrate mucosal immune responses and tolerance. These interactions are bidirectional: host diet, mucus, bile acids, immune activity, transit time, medication use, and disease state also shape the microbial community. Dysbiosis therefore describes a context-dependent departure from a reference community rather than a single diagnostic state. Although microbiome profiling may eventually support patient stratification, current measurements are not sufficiently standardized or disease-specific to guide DISH diagnosis or treatment [24].

## 11. Determinants of Gut Microbiota Composition

Gut microbiota composition changes in response to diet, antibiotics, gastrointestinal infection, age, geography, medication exposure, physical activity, sleep, and host physiology [93]. Diets rich in diverse fermentable plant substrates may support fiber-utilizing organisms, whereas low-fiber dietary patterns can reduce substrate availability for these communities. Antibiotics can cause marked losses or expansions of susceptible taxa, but the magnitude and recovery pattern vary by drug, dose, duration, and individual baseline. Stress-related neuroendocrine and motility changes may also influence the intestinal environment through the gut–brain axis [94]. These determinants frequently occur together and can confound disease comparisons. Dysbiosis has been reported in obesity, type 2 diabetes, irritable bowel syndrome, inflammatory bowel disease, and some neuropsychiatric conditions, but association does not identify whether the microbial change is a cause, consequence, treatment effect, or correlate of disease. Diet, selected prebiotics or probiotics, and other interventions can modify community features; FMT should not be described as a general treatment for dysbiosis and is clinically established only for limited indications [26,95].

These determinants are also important methodological confounders. Any comparison of gut microbiota in DISH should account for age, sex, geographic setting, dietary pattern, body mass index, diabetes, medication exposure—including antibiotics and proton-pump inhibitors—physical activity, and coexisting gastrointestinal disease. Without such adjustment, an apparent DISH-associated microbiome signature may instead reflect the clinical characteristics of the study population.

## 12. Microbiome Composition and Pathological Bone Formation

Most research on the microbiome–bone relationship has examined bone loss, fracture risk, or osteoporosis rather than pathological bone formation. For DISH, the relevant question is whether microbial or barrier-derived signals could favor osteoblast differentiation, entheseal mineralization, or ligament ossification in a metabolically susceptible host. Candidate pathways include low-grade systemic inflammation, altered bile-acid and SCFA signaling, endotoxin exposure, insulin and IGF-1 signaling, and immune mediators that regulate osteoblast–osteoclast coupling [96,97]. These pathways are biologically plausible but can produce different effects according to tissue, timing, concentration, and host phenotype. Evidence that preserves bone density cannot be assumed to explain hyperostosis, and an association between a microbial taxon and osteoporosis should not be transferred directly to DISH. Disease-specific studies should therefore measure ossification outcomes and pro-osteogenic signaling rather than relying only on bone-density endpoints.

## 13. Intervertebral Disc (IVD) Degeneration (IDD) and Gut Inflammation

Intervertebral-disc degeneration (IDD) and intestinal inflammation may share systemic inflammatory and metabolic influences, although direct communication between the gut and an intervertebral disc has not been established. Experimental and genetic association studies have proposed that barrier dysfunction, circulating microbial products, cytokines, and altered microbial metabolites could modify disc-cell stress, extracellular matrix turnover, or pain pathways [98,99]. Gut inflammation may also correlate with medication exposure, reduced physical activity, dietary change, and metabolic disease, all of which can independently affect spinal health. A Mendelian-randomization association or a microbiome difference does not demonstrate microbial entry into the disc or prove that microbiota modification will prevent degeneration [100]. IDD is included here because it illustrates possible systemic pathways relevant to spinal disease; it should not be treated as equivalent to DISH, which is defined by entheseal and ligamentous ossification [101].

## 14. The Gut–Spine Axis

The term gut–spine axis describes proposed links between intestinal microbial ecology and spinal tissues through circulating metabolites, immune mediators, endocrine signals, barrier dysfunction, and host metabolism (Figure 2) [99]. In IDD research, these pathways have been considered in relation to disc-cell inflammation and extracellular-matrix degradation. For DISH, the more relevant endpoints are osteogenic differentiation, enthesis mineralization, and ligament ossification. Nutrient malabsorption alone is unlikely to account for these changes, and evidence from osteoporosis or disc degeneration cannot be assumed to explain excessive bone formation. Studies of this axis must also control for age, obesity, diabetes, diet, activity, and medication exposure because each can influence both the microbiome and spinal outcomes [102].

Systemic inflammation and microbial metabolites may influence pain sensitization or musculoskeletal tissue responses, but these effects are not specific to DISH [103]. Current gut–spine evidence is derived mainly from experimental models, cross-sectional microbiome studies, and genetic-instrument analyses of related spinal phenotypes. It therefore provides hypotheses for mechanistic testing rather than a basis for clinical treatment. No study has shown that microbiome modification alters the initiation, rate of progression, or symptomatic burden of DISH.

## 15. Gut–BoneAxis: Osteoporosis and Hyperostotic Diseases

The gut–bone axis encompasses microbial effects on mineral absorption, immune regulation, endocrine signaling, and bone-cell activity [104]. Much of the evidence concerns osteoporosis, in which inflammatory and hormonal changes can shift remodeling toward bone loss. DISH presents a different biological problem: excessive ossification of ligaments and entheses. Plausible microbiota-related contributors to hyperostosis include metabolic endotoxemia, chronic low-grade inflammation, altered SCFA or bile-acid signaling, and insulin/IGF-1 pathways that may interact with osteogenic programs. Human DISH tissue studies have implicated pro-osteogenic signaling, including Wnt/β-catenin-related mechanisms, but these findings have not yet been connected directly to a gut-microbial exposure [105]. Accordingly, evidence that a microbial metabolite supports bone density should not be interpreted as evidence that it causes hyperostosis. Future work should compare DISH cases with metabolically matched controls and measure enthesis-specific osteogenic markers, circulating microbial metabolites, barrier integrity, and longitudinal imaging progression [106,107].

Reconciling osteoporosis evidence with ectopic ossification in DISH

A central interpretive problem is that most gut–bone studies focus on osteoporosis, a condition characterized by reduced bone mass and increased fracture susceptibility, whereas DISH is characterized by excessive ectopic ossification at ligaments and entheses. These processes are not simple opposites. A patient may have extensive spinal enthesophytes while trabecular bone quality is normal or reduced, and the rigid DISH spine may remain vulnerable to unstable fracture. Therefore, evidence that a microbial metabolite preserves systemic bone density cannot be assumed to explain, prevent, or promote the localized ossification characteristic of DISH [13,97].

Divergent skeletal outcomes may depend on concentration thresholds, duration of exposure, and differential expression of receptors for SCFAs or other microbial metabolites. Systemic signals may influence bone turnover throughout the skeleton, whereas the local mechanical and inflammatory environment of an enthesis may favor osteoblast differentiation and mineralization. At the same time, osteoclast activity, osteoblast activity, vascular invasion, and extracellular matrix remodeling may be regulated differently in trabecular bone, cortical bone, intervertebral tissues, and spinal ligaments. Thus, the same circulating metabolite could theoretically produce different effects across tissue compartments.

These possibilities remain mechanistic hypotheses. Direct DISH studies should compare circulating and local metabolite concentrations, receptor expression, osteogenic markers, osteoclast activity, and imaging progression. Until such evidence is available, osteoporosis studies should be used to identify candidate pathways, not as direct proof of a gut–DISH mechanism.

## 16. Gut–Joint Axis: Osteoarthritis (OA) and Facet Joint Syndrome

The proposed gut–joint axis is supported mainly by studies of osteoarthritis and inflammatory arthropathies rather than DISH [108,109]. Candidate pathways include systemic inflammation, barrier-derived microbial products, SCFAs and other metabolites, obesity-related mechanical loading, and immune regulation. Osteoarthritis involves cartilage and subchondral-bone changes, whereas DISH involves ligament and enthesis ossification; these are biologically distinct processes [1,108]. Findings from joint disease can therefore identify candidate inflammatory or metabolic pathways but should not be cited as direct evidence for a gut-microbiota–DISH mechanism. These proposed relationships and modifying factors are summarized in Figure 3.

## 17. Proposed Gut–Microbiota Pathways in DISH

A direct gut–DISH axis has not yet been demonstrated. DISH is a disorder of ligament and entheseal ossification rather than primary intervertebral-disc degeneration; associations involving IDD therefore identify possible shared systemic pathways but do not establish causation in DISH [110]. The rationale for investigation arises from the metabolic phenotype frequently accompanying DISH, including obesity, diabetes, hyperinsulinemia, and dyslipidemia, together with experimental evidence that microbial metabolites and inflammatory signals can influence host metabolism and bone-cell activity [111]. Barrier dysfunction or dysbiosis could, in principle, modify systemic cytokines, endotoxin exposure, bile acids, SCFAs, or endocrine signals that intersect with osteogenic pathways. However, nutrient absorption and generalized inflammation alone do not explain the characteristic flowing ossification. Fournier et al. assessed 1536 unique images comprising 16,710 motion segments and reported early-phase DISH in 13.2% of individuals and established DISH in 14.2%, with higher prevalence in males [112]. Such imaging phenotypes provide measurable outcomes for future longitudinal microbiome studies. Until those studies are performed, the proposed relationship should be presented as a mechanistic hypothesis rather than a recognized disease axis [113,114].

The gut microbiota can influence endocrine signaling in experimental models. In mice, microbial colonization has been associated with increased circulating insulin-like growth factor 1 (IGF-1) and bone growth [115]. IGF-1 participates in osteoblast function and skeletal remodeling, making this pathway relevant to a hypothesis about excessive ossification. Nevertheless, mouse growth and bone-formation findings cannot be extrapolated directly to the mature enthesis or ligament in human DISH. No study has shown that a microbiota-mediated change in IGF-1, insulin, or another hormone produces the characteristic flowing ossification of DISH. Human studies would need to relate microbial features and endocrine measurements to longitudinal imaging and tissue-level osteogenic markers before this mechanism could be considered causal.

## 18. Gut–Ligament Pathways and Lumbar Spinal Stenosis

The proposed gut–ligament relationship is based on indirect evidence from systemic inflammation, nutrient and endocrine signaling, and studies of spinal stenosis [99,100]. Spinal stenosis is anatomically and biologically distinct from DISH, although ligament hypertrophy or ossification can contribute to canal narrowing in some patients. Microbiota-associated changes in cytokines, insulin signaling, or mineral metabolism might modify connective-tissue remodeling, but these pathways have not been shown to initiate ligament ossification in DISH. Mendelian-randomization analyses can identify genetically instrumented associations, yet they remain sensitive to instrument validity, pleiotropy, population structure, and phenotype definition. In the cited spinal-stenosis study, the reported microbial associations did not remain significant after false-discovery-rate correction [100]. Direct evidence linking gut microbiota to DISH ligament or enthesis ossification is lacking, and observations from spinal stenosis should therefore be treated as hypothesis-generating.

## 19. Gut–Muscle Pathways and Spinal Sarcopenia

The gut–muscle axis concerns microbial and host pathways that may affect muscle mass, function, and recovery. Dietary protein and energy intake, physical activity, inflammation, microbial metabolites, and endocrine signals such as IGF-1 can interact in the development of sarcopenia [116,117,118,119]. In people with spinal disease, reduced paraspinal muscle mass may worsen mobility, balance, frailty, and rehabilitation outcomes. These consequences are clinically relevant to symptomatic DISH because stiffness, pain, and fracture risk can reduce activity. However, spinal sarcopenia does not explain the initiation of ligament or entheseal ossification, and no microbiome intervention has been shown to improve muscle or spinal outcomes specifically in DISH [49].

## 20. Potential Microbiota-Related Influences on DISH Pain

Pain in DISH can arise from stiffness, altered biomechanics, enthesopathy, fracture, or compression of adjacent structures. Gut microbial metabolites and immune signals can influence systemic inflammation and nociceptive processing in experimental or non-DISH clinical contexts [120,121]. The gut–brain axis also provides a route through which intestinal signals may modify pain perception, stress responses, and behavior [18]. These observations justify investigation but do not establish a microbiota-specific cause of DISH pain. No DISH study has shown that microbiome composition predicts pain severity or that prebiotics, probiotics, diet, or FMT relieves DISH-related pain. Future studies should use defined pain outcomes and control for structural disease burden, obesity, diabetes, medication use, sleep, mood, and physical activity [122].

## 21. Fecal Microbiota Transplantation and DISH

Fecal microbiota transplantation (FMT) transfers screened donor stool or a defined microbiota-based preparation to a recipient’s gastrointestinal tract. Current guideline-supported use is principally for selected patients with recurrent Clostridioides difficile infection [123]. Research in other gastrointestinal, metabolic, neurological, and immune conditions remains indication-specific and should not be generalized to DISH [26,57]. There is no preclinical or clinical evidence that FMT prevents ligament ossification, slows radiographic progression, or relieves pain in DISH. FMT also carries risks, including transmission of infectious organisms and other donor-associated traits, and requires regulated donor screening and clinical oversight. It should therefore not be proposed as a DISH treatment outside a rigorously justified research program.

At present, FMT is not an established treatment for DISH. No clinical trial has defined its safety, efficacy, donor selection, dosing, durability, or clinically meaningful outcomes in this population. Any application to DISH should therefore be confined to appropriately approved research and should not be inferred from its use in recurrent Clostridioides difficile infection or other gastrointestinal conditions.

## 22. Current Evidence, Limitations, and Research Priorities

The present evidence base supports biological plausibility rather than a confirmed gut-microbiota–DISH relationship. Direct DISH-specific microbiome studies are scarce. Most of the available argument is assembled from four related bodies of literature: the association of DISH with obesity, diabetes, and metabolic syndrome; experimental work on microbial metabolites and bone-cell regulation; observational and genetic studies of the gut microbiota in intervertebral disc degeneration or spinal stenosis; and broader studies of systemic inflammation, pain, and musculoskeletal health. These sources identify candidate mechanisms, but none alone demonstrates that dysbiosis precedes or causes the ligamentous ossification of DISH.

The first priority is a carefully phenotyped case–control study using accepted imaging criteria and matched controls. Age, sex, ethnicity, body mass index, diabetes, metabolic syndrome, diet, medication exposure, gastrointestinal disease, smoking, and physical activity should be measured as potential confounders. Microbiome profiling should be combined with targeted and untargeted metabolomics, inflammatory markers, and standardized radiographic measures. Replication in an independent population would be necessary before any microbial signature could be considered DISH-associated.

Longitudinal studies are then needed to establish temporal order. Serial imaging could determine whether microbial or metabolite profiles are associated with the transition from early entheseal changes to mature bridging, while fracture, dysphagia, pain, and functional outcomes would provide clinically meaningful endpoints. Mechanistic studies should test candidate metabolites at physiologically relevant concentrations in ligament- or enthesis-derived cells and examine receptor expression, osteogenic differentiation, osteoclast signaling, and the influence of mechanical loading. Only after consistent observational and mechanistic evidence is obtained would controlled microbiome-directed intervention studies be justified.

These requirements also define the present translational boundary. Routine microbiome testing cannot yet diagnose or prognosticate DISH, and probiotics, prebiotics, specialized diets, or FMT cannot currently be recommended as disease-modifying treatments. Their discussion in this review is intended to identify research directions and not to replace established clinical evaluation, fracture precautions, dysphagia assessment, rehabilitation, analgesia, or surgical management when indicated.

## 23. Conclusion and Perspective

The gut microbiota influences nutrient processing, SCFA and bile-acid metabolism, intestinal-barrier integrity, immune regulation, and systemic metabolic signaling. These functions provide a coherent biological framework for asking whether gut-derived signals may interact with the metabolic and inflammatory environment associated with DISH. The proposed gut–spine, gut–bone, gut–disc, gut–ligament, and gut–muscle axes are useful for organizing this question, but the strength of evidence differs considerably across them. Current evidence is strongest for the metabolic phenotype of DISH and for microbiome relationships with neighboring musculoskeletal conditions; it remains weakest for a direct causal connection between gut microbiota and DISH itself.

Future research should therefore move from broad analogy to disease-specific testing. Well-designed, longitudinal studies should determine whether microbial or metabolite signatures are reproducible, precede radiographic progression, remain significant after adjustment for metabolic confounders, and correspond to clinically important outcomes. Experimental work must also address the osteoporosis–hyperostosis paradox by defining how local tissue context, receptor expression, concentration thresholds, and osteoblast–osteoclast interactions shape the skeletal response to microbial metabolites. Until these questions are resolved, microbiome-directed interventions remain investigational. A more precise understanding of these pathways may ultimately identify biomarkers or therapeutic targets, but current clinical management should continue to rest on established diagnostic, metabolic, rehabilitative, and surgical principles.

## Figures and Tables

**Figure 1:**
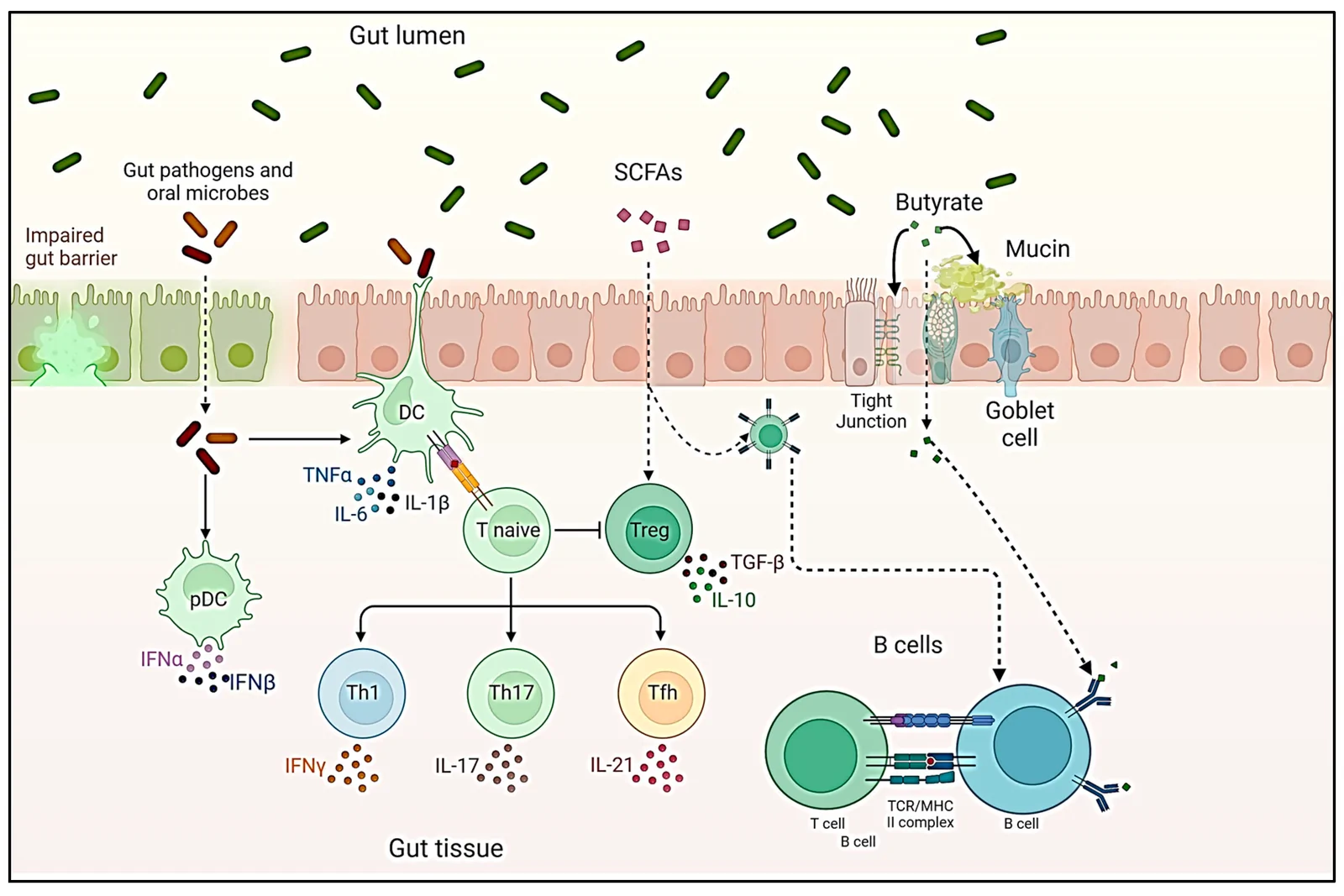
Gut-microbiota pathways involved in immune tolerance, intestinal-barrier integrity, and systemic inflammation. Microbial metabolites, including short-chain fatty acids, may influence dendritic-cell function, regulatory T-cell responses, B-cell signaling, tight-junction integrity, and cytokine production. The figure provides mechanistic context for investigating DISH but does not establish a direct causal relationship. Colors distinguish the illustrated cell and molecule types and do not encode quantitative values or effect direction. Abbreviations: SCFAs, short-chain fatty acids; DC, dendritic cell; pDC, plasmacytoid dendritic cell; Treg, regulatory T cell; Th1, T helper 1 cell; Th17, T helper 17 cell; Tfh, T follicular helper cell; TNF-α, tumor necrosis factor alpha; TGF-β, transforming growth factor beta; IL, interleukin; IFN, interferon; TCR, T-cell receptor; MHC II, major histocompatibility complex class II.

**Figure 2:**
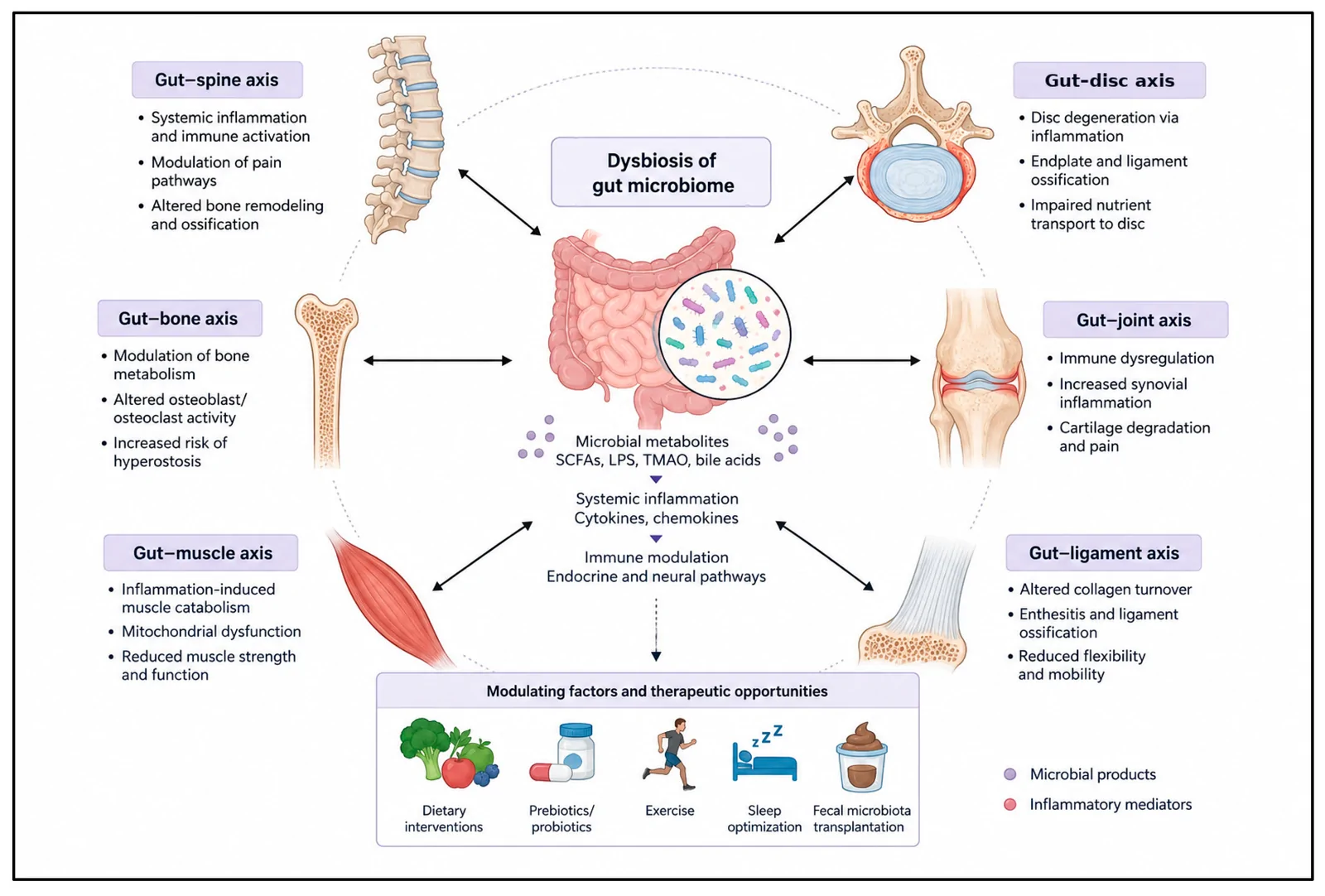
Conceptual relationships between gut-microbiota dysbiosis and spinal or musculoskeletal pathways. The arrows summarize proposed communication through microbial metabolites, intestinal-barrier dysfunction, systemic inflammation, cytokines, chemokines, immune modulation, and endocrine or neural signaling. They do not establish causation or treatment efficacy. Abbreviations: DISH, diffuse idiopathic skeletal hyperostosis; GM, gut microbiota; SCFAs, short-chain fatty acids; LPS, lipopolysaccharide; TMAO, trimethylamine N-oxide; IBD, inflammatory bowel disease; IBS, irritable bowel syndrome; IDD, intervertebral disc degeneration.

**Figure 3:**
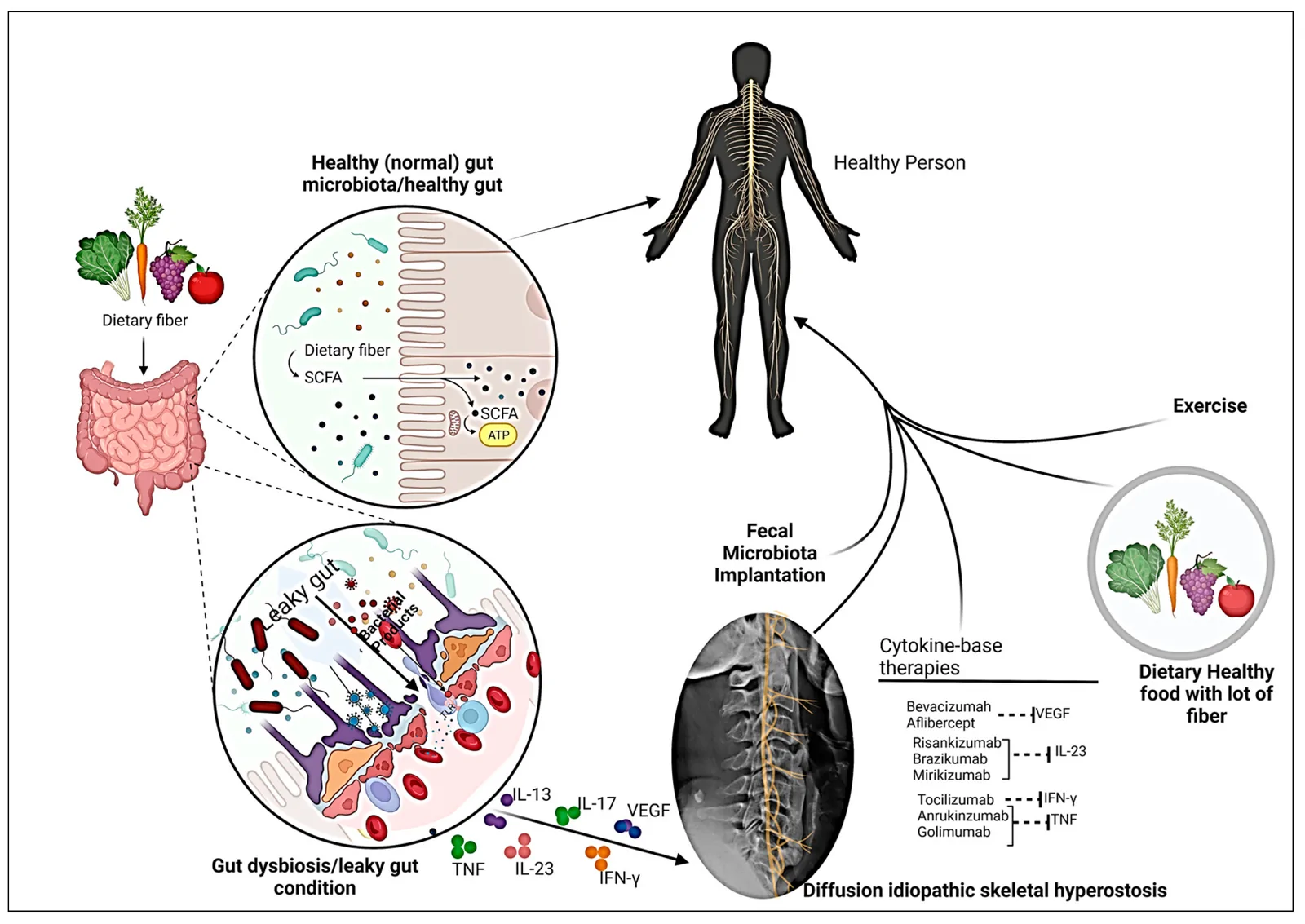
Hypothesized relationship between gut microbiota and DISH, with possible modifying factors. The diagram summarizes proposed roles for dysbiosis, inflammatory signaling, diet, exercise, and fecal microbiota transplantation. Colors distinguish the labeled cytokines, and arrows indicate hypothesized directional relationships or interventions; they do not establish causation or treatment efficacy. These relationships remain research hypotheses rather than established causes or treatments. Abbreviations: DISH, diffuse idiopathic skeletal hyperostosis; IL-13, interleukin-13; IL-17, interleukin-17; VEGF, vascular endothelial growth factor; IFN-γ, interferon gamma; TNF, tumor necrosis factor; IL-23, interleukin-23; FMT, fecal microbiota transplantation.

**Table 1:** Predicted biotransformation of eight common short-chain fatty acid input compounds. All eight input compounds are included; methanoic acid yielded no predicted product under the specified settings. USGBE, unspecified gut bacterial enzyme. The entries are unvalidated in silico predictions generated by submitting the SCFA SMILES to the BioTransformer web server (accessed in September 2023; exact server build was not retained). They do not demonstrate gut or host metabolism in vivo.

Input Compound	Enzymes	Reaction Type	Biotransformation Reaction
Methanoic acid	—	No product predicted	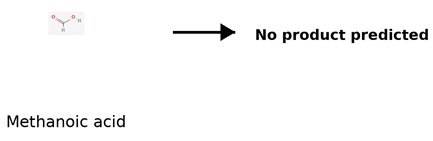
Ethanoic acid	Bacterial UDP-glucuronosyltransferase	O-Glucuronidation of aliphatic acid	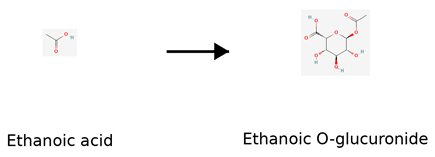
Propanoic acid	Unspecified gut bacterial enzyme	α-Oxidation of carboxylic acid	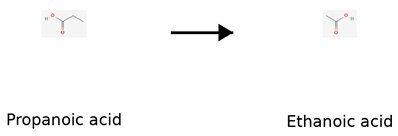
Propanoic acid	Bacterial UDP-glucuronosyltransferase	O-Glucuronidation of aliphatic acid	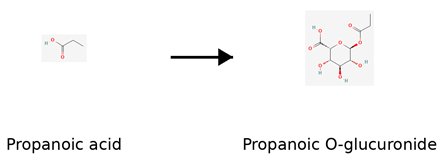
Butanoic acid	USGBE	β-Oxidation of carboxylic acid	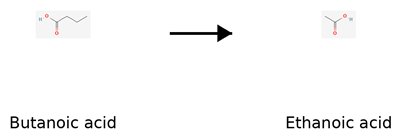
Butanoic acid	Bacterial UDP-glucuronosyltransferase	O-Glucuronidation of aliphatic acid	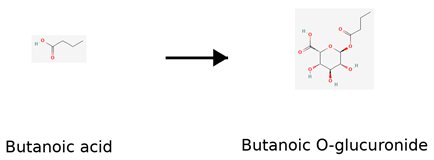
2-Methylpropanoic acid	Bacterial UDP-glucuronosyltransferase	O-Glucuronidation of aliphatic acid	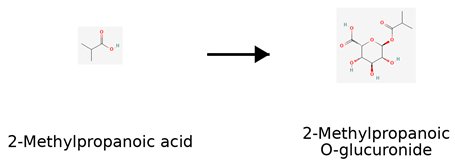
Pentanoic acid	Unspecified gut bacterial enzyme	β-Oxidation of carboxylic acid	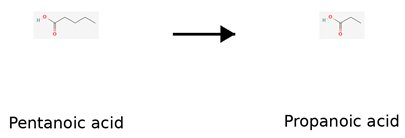
Pentanoic acid	Bacterial UDP-glucuronosyltransferase	O-Glucuronidation of aliphatic acid	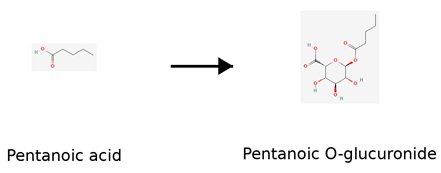
3-Methylbutanoic acid	Unspecified gut bacterial enzyme	α-Oxidation of carboxylic acid	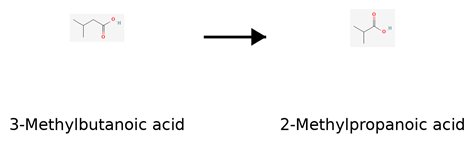
3-Methylbutanoic acid	Bacterial UDP-glucuronosyltransferase	O-Glucuronidation of aliphatic acid	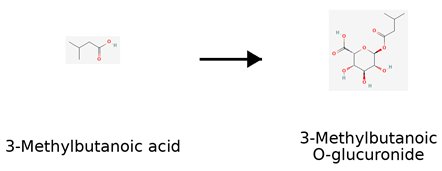
2-Methylbutanoic acid	Bacterial UDP-glucuronosyltransferase	O-Glucuronidation of aliphatic acid	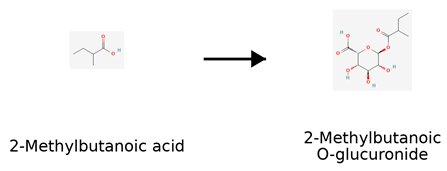

**Table 2:** Selected cytokines and growth factors involved in immune regulation and tissue homeostasis, with representative responding cells, disease contexts, and functions. The cell column lists targets of signaling rather than cytokine-producing cells and is not exhaustive. Disease contexts and functions reflect the cited literature across intestinal and other tissues; they do not establish causal roles in DISH. Effects depend on receptor expression, tissue, timing, and interactions with other mediators.

No.	Cytokine	Representative Responding Cells	Reported Disease Contexts	Representative Functions	Refs
**1.**	**Interleukin-10 (IL-10)**	Monocytes, Mφ, dendritic cells	Inflammatory bowel disease, autoimmunity, allergy, asthma, cancer	Anti-inflammatory cytokine Regulates immune responses. Maintains intestinal homeostasis by suppressing excessive immune activation. Protective role against colitis and other inflammatory bowel diseases	**[70]**
**2.**	**Vascular Endothelial Growth Factor (VEGF)**	Intestinal microvascular endothelial cells	Inflammatory bowel disease	VEGF-A promotes angiogenesis and inflammatory activation of intestinal microvascular endothelial cells.	**[71]**
**3.**	**Tumor Necrosis Factor-alpha (TNF-α)**	Immune cells, endothelial cells, fibroblasts	Rheumatoid arthritis, inflammatory bowel disease, psoriasis, ankylosing spondylitis	Induces inflammation and cell death. Implicated in Crohn’s disease and ulcerative colitis. Target of anti-TNF therapies in IBD	**[72]**
**4.**	**Amphiregulin (Areg)**	Epithelial cells, fibroblasts, Tregs	Inflammatory tissue injury, cancer	Areg is an epidermal growth factor involved in cell growth, proliferation, differentiation, and tissue repair.	**[73]**
**5.**	**Interleukin-6 (IL-6)**	Hepatocytes, monocytes, Mφ, neutrophils, T-cell subsets	Rheumatoid arthritis and associated systemic manifestations	IL-6 regulates acute-phase responses and promotes inflammatory signaling; classical and trans-signaling affect different cell populations.	**[74]**
**6.**	**Oncostatin M (OSM)**	Neurons, astrocytes, endothelial cells	Neuroinflammatory and neurodegenerative disorders	OSM modulates inflammation, cell survival, and tissue repair, with context-dependent effects in the nervous system.	[75]
**7.**	**Interleukin-12 (IL-12)**	NK cells, T cells	Infectious and autoimmune inflammatory diseases	Pro-inflammatory cytokine. Enhances cell-mediated immune responses Promotes Th1 differentiation Implicated in Crohn’s disease	**[76]**
**8.**	**Interleukin-15 (IL-15)**	NK cells, memory CD8+ T cells	Autoimmune inflammatory diseases, cancer	IL-15 is primarily involved in the proliferation, activation, and survival of various immune cells, particularly natural killer (NK) cells and memory CD8+ T cells.	**[77]**
**9.**	**Interleukin-17 (IL-17)**	Epithelial cells, stromal cells	Cancer and chronic inflammatory disorders	IL-17 promotes inflammatory signaling, recruitment of myeloid cells, and epithelial or stromal responses; effects vary by tissue and disease context.	**[78]**
**10.**	**Interleukin-11 (IL-11)**	Megakaryocyte precursors	Fibrosis, cancer; thrombocytopenia (therapeutic context)	IL-11 supports megakaryocyte maturation and platelet production and also participates in inflammation, fibrosis, and cancer.	**[79]**
**11.**	**Interferon-gamma (IFN-γ)**	Mφ, hematopoietic progenitor cells	Hemophagocytic lymphohistiocytosis, aplastic anemia	IFN-γ activates macrophages and antimicrobial responses; excessive signaling contributes to inflammatory pathology and suppression of hematopoiesis.	**[80]**
**12.**	**Interleukin-22 (IL-22)**	Epithelial cells	Pulmonary inflammation and infection	IL-22 acts on epithelial tissues to regulate barrier defense and repair; its effects depend on the inflammatory context.	**[81]**
**13.**	**Interleukin-2 (IL-2)**	Activated T cells, Tregs, NK cells	Autoimmune disorders, cancer	IL-2 supports activated T-cell proliferation and regulatory T-cell maintenance, contributing to both immunity and tolerance.	**[77]**
**14.**	**Interleukin-23 (IL-23)**	Th17 cells	Crohn’s disease, psoriasis	IL-23 supports expansion, maintenance, and inflammatory functions of Th17 cells and contributes to immune-mediated disease.	**[82]**
**15.**	**Granulocyte-Macrophage Colony-Stimulating Factor (GM-CSF)**	Myeloid progenitors, neutrophils, Mφ	Periodontitis, rheumatoid arthritis	GM-CSF is primarily involved in stimulating the production, maturation, and activation of white blood cells, including granulocytes and macrophages.	**[83]**
**16.**	**Transforming Growth Factor-beta (TGF-β)**	T cells, B cells	Autoimmune disease, cancer	Has both pro-inflammatory and anti-inflammatory functions. Regulates immune cell differentiation Involved in immune tolerance and tissue repair. Balances inflammatory responses	**[84]**
**17.**	**Interleukin-7 (IL-7)**	T cells, lymphoid progenitors	Bacterial respiratory infection (experimental models)	IL-7 supports lymphocyte development and survival and enhances protective innate T-cell responses in experimental respiratory infection.	**[85]**
**18.**	**Interleukin-1 (IL-1)**	Myeloid cells, endothelial cells, fibroblasts	Autoinflammatory syndromes, gout	Pro-inflammatory cytokine. Induces inflammation and fever Plays a role in gut inflammation and mucosal responses. Implicated in IBD and other gut disorders	**[86]**
**19.**	**Interleukin-33 (IL-33)**	Mast cells, Th2 cells, ILC2s	Sjögren’s syndrome	IL-33 acts as an alarmin through ST2 and modulates inflammation, type 2 immune responses, and tissue repair.	**[87]**
**20.**	**Interleukin-4 (IL-4)**	B cells, Mφ, epithelial cells	Gastric and colon cancer, allergic disease	IL-4 promotes type 2 immunity, B-cell responses, and alternative macrophage activation; it can contribute to allergy and context-dependent tumor responses.	**[88]**
**21.**	**Interleukin-5 (IL-5)**	Eosinophils	Severe eosinophilic asthma	IL-5 is primarily associated with the regulation and activation of eosinophils, a type of white blood cell involved in the immune response to parasitic infections and allergic reactions.	**[89]**
**22.**	**Interleukin-9 (IL-9)**	Monocytes and osteoclast precursors	Rheumatoid arthritis	IL-9 promotes osteoclast differentiation in experimental studies using cells from patients with rheumatoid arthritis.	**[90]**
**23.**	**Interleukin-13 (IL-13)**	Mφ, epithelial cells, fibroblasts	Gastric and colon cancer, allergic disease	IL-13 regulates type 2 responses and epithelial or stromal activity; it can contribute to allergy, tissue remodeling, and context-dependent tumor responses.	**[88]**
**24.**	**Interleukin-25 (IL-25)**	ILC2s, Th2 cells	Infectious and allergic diseases	IL-25 is primarily involved in regulating immune responses, particularly in promoting type 2 immune responses, which are associated with allergies and immunity against parasitic infections.	**[91]**
**25.**	**Thymic Stromal Lymphopoietin (TSLP)**	Dendritic cells, T cells, B cells, ILCs	Asthma, atopic dermatitis	TSLP is a cytokine that is primarily involved in regulating immune responses, particularly in the context of allergies and inflammatory conditions.	**[92]**

Abbreviations: Mφ, macrophages; NK, natural killer; ILCs, innate lymphoid cells; ILC2s, group 2 innate lymphoid cells; Tregs, regulatory T cells; Th2 and Th17, T helper 2 and 17 cells, respectively; ST2, the IL-33 receptor.

## Data Availability

The literature supporting this review is cited throughout the manuscript. The biotransformation predictions generated using the BioTransformer web server (accessed in September 2023; exact server build was not retained) are presented in Table 1, and the archived uncleaned computational output underlying Table 1 is provided in Supplementary Data S1. These predictions were not experimentally validated, and no new experimental data were generated.

## Supplementary Materials

The following supporting information can be downloaded at: https://www.mdpi.com/article/10.3390/nu18193168/s1, Supplementary Data S1 contains the archived uncleaned computational output underlying Table 1.
